# New Diagnostic Strategy for Onychomycosis: First-Line Utilization of DermaGenius^®^ PCR and Calcofluor Microscopy Combined with Selective Culturing

**DOI:** 10.3390/jof10080515

**Published:** 2024-07-24

**Authors:** Séverine Evrard, Caroline Minon, Mouhsine Lamtiri Laarif, Benjamin De Backer, Henry Paridaens, Marie-Pierre Hayette, Julie Frère, Jean-Marc Senterre, Jean-Marc Minon

**Affiliations:** 1 Laboratory Medicine Department, Centre Hospitalier Régional de la Citadelle, 4000 Liege, Belgium; caroline.minon@citadelle.be (C.M.);; 2 Department of Clinical Microbiology, Centre Hospitalier Universitaire du Sart-Tilman, 4000 Liege, Belgium; 3 Pediatrics Department, Centre Hospitalier Régional de la Citadelle, 4000 Liege, Belgium

**Keywords:** onychomycosis, dermatophytes, mold, yeast, calcofluor, hyphae, diagnostic strategy, multiplex PCR, culture

## Abstract

Onychomycosis (OM) is a widespread infection requiring prolonged treatment with potential side effects. Diagnostic certainty is therefore essential before initiating antifungal therapy. Molecular biology has already shown benefits in reducing the time to diagnosis, providing technical ease, and increasing sensitivity for the respective species that molecular tests can detect. Nevertheless, causative agents are numerous, and culture remains essential, particularly for detecting non-dermatophytes mold infections. This study compared the performance of three different diagnostic strategies: conventional culture technique, the multiplex DermaGenius^®^ 2.0 PCR (DG), and a mixed PCR/culture algorithm guided by the result of direct examination with calcofluor (DEC). The mixed algorithm (MA) prioritizes DG PCR and DEC as the primary diagnostic tools, supplemented by selective sample inoculation when mycelial elements are visualized in DEC and when DG PCR fails to detect any fungus or identifies a fungus with morphology differing from that observed in DEC (filamentous fungi versus yeasts). With only 13% of samples requiring inoculation, MA emerged as the most effective strategy, demonstrating significantly higher sensitivity (98.18%; *p* < 0.001) compared to single-method approaches (78.18% for DG PCR alone and 74.55% for culture alone) while maintaining a specificity comparable to DG PCR (100%). This new approach saves time in result delivery, requires fewer human resources, and increases diagnostic accuracy to better meet the needs of clinicians.

## 1. Introduction

Onychomycosis (OM) is a frequent fungal infection whose prevalence increases with age and is estimated at 10–15% in the world population [1,2]. Predisposing factors include alterations in the peripheral circulatory system, psoriasis, repeated trauma, diabetes, hemodialysis, and immunosuppression [1,3,4].

OM is mainly caused by keratolytic dermatophyte fungi (*Tinea unguium*), but non-dermatophytes molds (NDM) and yeasts may also be involved. NDM cause between 2% and 12% of OM, depending on population and geographical conditions [1]. As they are filamentous fungi of environmental origin, NDM may sometimes contaminate samples and/or culture, the frequency of which varies with species. To ascertain their pathogenicity, diagnostic criteria such as visualization of fungal elements in direct examination and/or isolation of NDM from a significant number of inocula have historically been proposed [1,5]. Mixed dermatophyte–NDM infections can occur in 3–11% of OM, with the clinical significance of NDM still poorly understood in this scenario [6]. Yeasts are the third cause of OM, and their detection is predominantly associated with immunosuppression or diabetes [2,7].

Clinical manifestations of OM may be nonspecific, necessitating the exclusion of several differential diagnoses [8]. Treatment of OM is long and can cause side effects, particularly when systemic therapy is required. Prior to its initiation, it is therefore imperative to confirm the diagnosis through reliable and ideally rapid laboratory analysis [8,9,10,11]. Epidemiological studies carried out in Europe and the United States still report the widespread use of empirical treatment (without sampling) [12,13], a practice potentially influenced by the suboptimal fulfillment of clinicians’ needs by conventional techniques such as direct examination and culture. Direct examination fails to accurately identify the assaulting fungal species, which is crucial for selecting appropriate treatment, while culture lacks sensitivity and necessitates 3 to 4 weeks until the causative agent is confirmed [14]. To overcome culture’s limitations, laboratories are showing increasing interest in molecular diagnostic techniques, whether in-house or commercial [9,10,15,16,17,18], which have demonstrated efficacy in expediting diagnosis (48 h versus 1 to 4 weeks for culture), ease of use, and enhanced sensitivity for targeted species. Their performance is nevertheless limited by the number of species targeted (most often dermatophytes), and culture currently remains the most suitable technique for screening NDM and/or yeast infections (excluding *C. albicans*). Molecular biology and culture techniques can therefore be seen as complementary.

DermaGenius^®^ 2.0 (DG) (PathoNostics B.V, Maastricht, The Netherlands) is a commercial multiplex PCR kit designed for the detection of *C. albicans* and 11 dermatophytes. Our study assessed the performance of DG multiplex PCR compared with conventional culture technique and the relevance of a mixed strategy, henceforth termed the “mixed algorithm” or MA, which prioritizes DG PCR as the primary diagnostic tool, supplemented by selective culture of specific samples. Agar inoculation is exclusively applied either when direct examination with calcofluor (DEC) reveals mycelial elements, but DG PCR is negative, or when both DG PCR and DEC are positive for fungi of different types (filamentous fungi versus yeasts). In other scenarios, definitive response relies solely on DG PCR, with a maximum delay of one week.

## 2. Materials and Methods

The study was conducted at Citadelle Hospital between the 1st January 2023, and the 1st May 2023. For all nail specimens received during this period in the laboratory, DEC, culture, and DermaGenius^®^ 2.0 multiplex PCR were systematically performed according to the procedures described below. For each sample, demographic data including patient age and gender, type of prescriber, community living, and nail location (hand, foot, or unspecified) were collected. Samples too small to carry out both techniques in parallel were excluded. All samples were provided as part of a non-interventional standard-of-care diagnostic procedure by a qualified medical diagnostic laboratory, and as such, they do not represent a clinical trial for which an ethics overview and informed consent are required.

- **Direct examination with calcofluor (DEC)**

A fragment of each nail was placed on a slide (Epredia, Breda, The Netherlands) and recovered by two drops of 20% KOH solution. The preparation was covered with a coverslip and placed in a humidity chamber at room temperature overnight. The following day, the coverslip was lifted, and a drop of Calcofluor White^®^ (Becton Dickinson, East Rutherford, NJ, USA) was applied to the disaggregated sample. The mounted slide was examined under fluorescence microscopy for the presence of mycelial elements (yeasts, pseudohyphae, hyphae, or arthrospores).

- **Culture**

Small nail fragments (5 to 6 per agar) were embedded in two types of agar tube: Sabouraud chloramphenicol agar (Biomérieux, Marcy-l′Étoile, France) and Sabouraud chloramphenicol agar with cycloheximide (Biomérieux, Marcy-l′Étoile, France). These agars were then incubated at 28(+/−2) °C for 4 weeks and examined weekly by an experienced operator. In the event of fungal growth, the different morphotypes were subcultured on potato glucose agar (Becton Dickinson, East Rutherford, NJ, USA), which promotes sporulation. Identification was then carried out on the basis of the macroscopic and microscopic characteristics, except for fungi with a yeast-like appearance, for which the MALDI-TOF MS technique was used (installed version: 4.2.100.19). If filamentous fungi could not be identified via macroscopic/microscopic examination, the strain was referred to the reference center at Liège University Hospital for identification using sequencing techniques.

- **PCR DermaGenius^®^ 2.0 (PathoNostics B.V., Maastricht, The Netherlands)**

The DG technique was performed according to the manufacturer’s specifications. A nail fragment was placed in a vial containing 650 µL of proteinase K and incubated overnight at 56 °C. The preparation was then vortexed at 1400 rpm and briefly centrifuged. DNA was then extracted from the supernatant using the automated E-Mag^®^ system (BioMérieux, Marcy-l′Étoile, France), with 15 µL of internal control (included in the kit) added to each sample. Then, 5 µL of DNA extract was added to two different PCR mixes (composed by 10 µL of primers and probes): 1.5 µL of Taq polymerase and 8.5 µL of dilution buffer. The amplification was performed using a LightCycler^®^ 480II (Roche, Bâle, Suisse). The targets of the Derma Genius 2.0 kit are defined as follows: *Trichophyton rubrum*, *Trichophyton interdigitale*, *C. albicans*, *Trichophyton tonsurans*, *Trichophyton mentagrophytes*, *Trichophyton soudanense*, *Trichophyton violaceum*, *Trichophyton benhamiae*, *Trichophyton verrucosum, Microsporum canis*, *Microsporum audouinii*, and *Epidermophyton floccosum*.

Negative and positive controls were incorporated into each run. Positive results were assessed by melting curve analysis, enabling specific identification of different dermatophyte species.

- **Mixed algorithm (MA)**

The mixed algorithm (MA) proposed first-line DG PCR implementation and selective inoculation of samples either when DEC revealed mycelial elements, but DG PCR was negative, or when both DG PCR and DEC were positive for fungi of different types (filamentous fungi versus yeasts).

Response was therefore based solely on DG PCR results and provided within a week in the following scenarios:

- Positive DEC with hyphae and detection of a dermatophyte on PCR;
- Positive DEC with pseudohyphae or yeast and detection of *C. albicans* on PCR;
- Both DEC and PCR are negative;
- Negative DEC and detection of a dermatophyte and/or *C. albicans* on PCR (uncommon situation; if applicable, as with *C. albicans*, response is provided with a comment on the fungus’s colonization potential).

In other cases, culture was performed secondarily, with responses provided within one to four weeks:

- Positive DEC (whatever the type of fungal elements observed) and negative PCR;
- Positive DEC with yeasts/pseudohyphae and detection of only a dermatophyte on PCR (uncommon situation);
- Positive DEC with hyphae and detection of only *C. albicans* on PCR (uncommon situation).

1. **Interpretation of results**

All dermatophytes detected were considered as proven pathogens regardless of the detection circumstances. Each other situation involving NDM or yeast was assigned to a clinical category, considering the intrinsic pathogenic potential of the fungus in OM, DEC positivity, and possible co-detection of a fungus of greater clinical significance.

Clinical categories and their arbitrary definitions are presented in Table 1, while Appendix A lists the fungal species detected, their occurrence, and the clinical categorization assigned to each situation.

In order to effectively compare performance of the different strategies (multiplex PCR alone, culture alone, or “mixed algorithm”) in terms of sensitivity and specificity in OM diagnosis, the results obtained by each of these methods were compared with the results obtained by combining the two complementary techniques (PCR and culture systematically associated), a strategy arbitrarily defined in this study as the new gold standard.

For sensitivity calculation, samples for which at least one fungal species could be categorized as a proven, probable, or possible pathogen were considered positive; samples containing only contaminants were considered negative.

Considering the excessive work involved in samples containing only contaminants, these were considered as false-positive samples in specificity calculation.

2. **Statistical analysis**

For each diagnostic strategy, the sensitivity, specificity, and a Cohen’s kappa coefficient (*K*) were calculated. The *K* measures the degree of agreement with the gold standard method and was interpreted as follows: ≤0: strong disagreement; 0–0.20: very weak agreement; 0.21–0.40: weak agreement; 0.41–0.60: average agreement; 0.61–0.80: satisfactory agreement; 0.81–1.00: excellent agreement. The different diagnostic strategies were compared with each other using the McNemar test. The significance threshold was set at 5% (*p* < 0.05). IBM SPSS Statistics software version 29.0.1.0 was used for calculations.

## 3. Results

A total of 106 samples from 102 patients were included in the study, with a median patient age of 55 years (ranging from 13 to 97 years) and a predominance of female patients (59.4%). When the location of the lesion was specified by the prescriber (43 samples out of 106), it mainly concerned toenails (95%). Requests for analysis came mainly from general practitioners (67%) or dermatologists (27%). Apart from one analysis for one hospitalized patient, all other requests came from ambulatory medicine, in which 7% of cases concerned patients living in institutions (nursing homes, psychiatric hospitals, or prisons).

Positivity was defined by the presence of at least one certain, probable, or possible pathogen in the sample. According to our newly established gold standard (combination of results obtained by DG PCR and culture), 55 samples (51.9%) were positive. DG PCR detected positivity in 43 samples (40.6%), with culture confirming the DG PCR results in these particular samples in only 62.8% of cases. Conversely, culture identified potential pathogens in 12 samples (11.3%) that were tested negative by DG PCR. Most of these specimens involved NDM (eight samples), and only one case involved a yeast (*C. parapsilosis*), but culture also recovered three dermatophytes that were not detected on the first PCR run.

Regarding the species of interest, *Trichophyton rubrum* and *Trichophyton interdigitale* were the sole dermatophytes identified, being present in 80% of positive samples either alone or in combination with mold or yeast. Coinfections were observed in 15% of positive samples, with a dermatophyte present in all instances. NDM alone were incriminated in 15% of positive cases, while yeasts alone were detected in 5% of them. Among the 14 NDM categorized as potential pathogens and detected either alone or in co-infection, we found five *Scopulariopsis*, three *Acremonium*, two *Fusarium*, one *Scytalidium dimidiatum*, one *Aspergillus versicolor*, one *Aspergillus flavus*, and one *Aspergillus fumigatus*. In terms of yeasts, only *Candida albicans* (two samples) and *Candida parapsilosis* (three samples) were recognized as potentially pathogenic.

**Figure 1:**
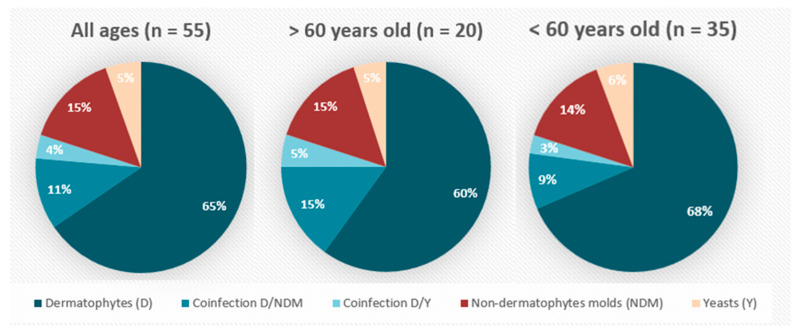
Distribution of fungus type according to the patient’s age.

Positivity did not differ significantly between patients under and over 60 years of age (53.8% versus 48.8%). Nevertheless, there was a trend towards a decrease in single dermatophyte infections and an increase in co-infections in people over 60 (Figure 1).

The performances of the different strategies assessed in OM diagnosis are summarized in Table 2 below.

If samples containing only possible pathogens were considered positive, the sensitivity of independent PCR or culture strategies were similar (78.18% for PCR and 74.55% for culture). With this calculation method, sensitivity of PCR was excessively worsened, as PCR showed a clear advantage when only proven or probable pathogens were taken into account (sensitivity of 82.69% for PCR and 67.31% for culture). In both situations, the MA strategy demonstrated significant superiority in comparison with the two other ones, i.e., sensitivity of 98.18% or 100.00%, depending on inclusion of possible pathogens in the calculation or not. The only positivity missed by MA concerned an NDM (*Fusarium* sp.) categorized as a possible pathogen due to its strong association with OM but a negative DEC on sample. Given the negative results of both DEC and DG PCR, this particular sample did not encounter criteria for culturing in the MA strategy.

The MA strategy demonstrated excellent agreement with the gold standard, as indicated by Cohen’s kappa (*K* = 0.98), while culture (*K* = 0.74) and DG PCR (*K* = 0.78) showed satisfactory agreements. When focusing solely on species detectable by the DG multiplex PCR DG, the sensitivity gap between PCR and culture strategies widened logically (sensitivity of 93.48% for PCR and 58.70% for culture). Nevertheless, it is noteworthy that the MA strategy even exhibited a trend to better detect these specific species than the PCR strategy itself, as a positivity was missed on the first PCR run for three samples and recovered from selective culturing. This discrepancy may be explained by the heterogeneity of the samples, counterbalanced in culture by plating several nail pieces. For these three samples, repetition of PCR analysis highlighted dermatophyte species that were previously missed.

Specificity was excellent for both the PCR and MA strategies, whereas it was poor for culture due to numerous contaminations.

The direct examination with calcofluor (DEC) yielded positive results in 52 samples. Among these, a pathogen was identified either by PCR or culture in 49 cases, while 3 remained positive without further characterization. Of the 55 samples classified as positive by our new gold standard, 49 tested positive on DEC. Among the six false-negative results, four were identified as positive by DG PCR for a dermatophyte without culture confirmation; one was positive by DG PCR for a dermatophyte, whereas an *Acremonium* spp. was identified as a possible pathogen in culture; and only one sample had a negative DG PCR, with *Fusarium* spp. in culture considered as a possible pathogen as well. Therefore, DEC demonstrated a sensitivity of 89.1% compared to our new gold standard (95.1% compared to culture) and a specificity of 97.2%.

Culture revealed a mixed infection in four samples, all involving the presence of a dermatophyte (three *Trichophyton rubrum* and one *Trichophyton interdigitale*); two of them were considered coinfected with *Candida parapsilosis*, one with *Scopulariopsis*, and the last one with *Acremonium*. As DEC of the two samples coinfected with *Candida parapsilosis* revealed yeasts in addition to hyphae, and the PCR result only indicated a dermatophyte, these two coinfections were also identified by MA, which recommends re-culturing in such cases. Finally, the *Trichophyton rubrum*/*Acremonium* coinfection was also identified by the mixed algorithm because this sample was among the three samples for which the first PCR run was negative, despite the presence of a dermatophyte. The DG PCR alone did not identify any coinfected samples, as multiple targets were never detected in the same sample. Based on this study, culture appears to be the most suitable strategy for identifying coinfections, followed by MA, whereas PCR alone is not an adequate method for their detection. However, this observation is based on a very limited number of cases.

## 4. Discussion

Identifying the pathogen in suspected cases of OM is crucial for optimal patient management. Even though OM is not a life-threatening infection, a rapid diagnosis is essential to prevent the infection from spreading, to ensure more effective and shorter treatments, and to avoid complications such as pain, nail deformities, and secondary bacterial infections. Early detection also helps maintain the patient’s quality of life by addressing aesthetic and psychological concerns. While confirming mycological diagnosis can be rapidly achieved through direct microscopy using fluorescence [19], identifying the infectious agent is equally important, as treatment varies depending on the type of fungus. For instance, NDM such as *Fusarium* spp. and *Aspergillus* spp. do not respond to standard treatments with terbinafine and itraconazole, typically used for dermatophytes infections [20].

The demographic data from our study are consistent with the literature. Only 1.9% of the samples were from children, confirming the rarity of OM in this group [2]. Lesions were mainly on toenails (95%), which aligns with the most common reported location of OM [2,21]. Although yeasts typically affect hands, and molds are more commonly associated with foot infections [22], our sample size was too small to analyze these epidemiological differences. However, age appears to influence the type of infecting fungi, with individuals over 60 showing a trend towards fewer dermatophyte-only infections and more co-infections, a phenomenon documented in the literature and linked to comorbidities [1]. On epidemiological aspects, the most frequently isolated dermatophyte species in our study, *T. rubrum* and *T. mentagrophytes* complex, are consistent with findings from Belgium [23] and other parts of Europe [24,25,26]. Similarly, the molds (*Scopulariopsis, Acremonium, Fusarium, Scytalidium dimidiatum,* and *Aspergillus* spp.) and yeasts (*Candida parapsilosis* and *Candida albicans*) categorized as pathogens in our study are among the most frequently reported non-dermatophytic species [1,7].

Several studies have shown that combining PCR and culture techniques reduces the false-negative rate in diagnosing onychomycosis [17,27,28,29]. Nonetheless, routinely implementing both methods is impractical due to the high cost of PCR reagents and the labor-intensive nature of culture. Although PCR improves dermatophyte detection and provides reliable diagnoses even with suboptimal samples [17] or previously treated patients [9], culture enables the identification of less frequently implicated fungi, predominantly NDM, which are often not targeted by PCR assays. Therefore, both techniques are complementary. This is why most authors agree that molecular tools should not fully replace conventional diagnosis but should be used alongside conventional mycological tools [17].

While the interest in PCR is now well documented, only a few studies have attempted to clearly define the role of PCR in routine workflow. Some authors suggested replacing direct examination with systematic PCR testing [30], but direct examination remains helpful to assess the pathogenicity of NDM [1], and its execution is easy and inexpensive. Burgos et al. [31] and Petinataud et al. [17] both suggested a new workflow, advocating for PCR to be conducted solely when KOH direct examination yields a positive result, possibly omitting culture when PCR returns positive [31], while culture is always performed on samples with a negative KOH direct examination or when PCR returns negative. This option allows to rapidly distinguish between NDM and dermatophytes infection, as PCR is highly effective in detecting dermatophyte, but the proportion of samples requiring culture remains high, representing over two-thirds of samples if this algorithm had been applied in our study. Petinataud et al. [17] also proposed another strategy that considers PCR as a second-line test performed after about a week of culture and only on samples resulting in negative cultures. Similarly, Pospischil et al. [28] preconized for adding molecular methods when conventional diagnostics are inconclusive. This approach would unfortunately partially lose one of the major advantages of PCR: its speed.

To reduce costs, enhance sensitivity, deliver faster results, and create a realistic workflow for routine laboratories, our study proposes a new algorithm that minimizes the number of cultured samples by using PCR and DEC results as first-line diagnostics. To our knowledge, this is the first study to evaluate an MA promoting sample inoculation solely in cases where DEC is positive and where DG PCR is negative for a morphologically compatible fungus. In all other scenarios, including samples with both negative DEC and PCR results, the final response is provided directly by DG PCR, conducted weekly (details outlined in the methodology section). MA was compared to more conventional approaches, such as using PCR or culture as singular methods. To overcome bias introduced by the lack of sensitivity of reference techniques (culture and KOH direct examination) [17], we compared these three strategies with a new gold standard, i.e., the results obtained by the systematic combination of a DG PCR and a culture. DEC was not considered as a standalone diagnostic strategy due to its inability to identify fungal species, which is crucial for treatment selection. However, its excellent performance, as previously described in numerous studies [17,32,33], was confirmed in our study with a sensitivity and specificity of 89.1% and 97.2%, respectively, compared to our new gold standard (sensitivity of 95.1% compared to culture only). Its ideal performance is harnessed in MA, which uses it as a sorting tool to decide which samples require additional culturing.

While in our study, the gain of sensitivity of the DG PCR technique over culture is undeniable for species it can detect (93.48% versus 58.70% for culture; *p* < 0.001), its overall sensitivity in diagnosing OM is not significantly different from that of culture (78.18% versus 74.55% for culture; *p* = 0.85) due to its inability to detect NDM and yeasts other than *C. albicans*. Nevertheless, DG PCR provides greater diagnostic certainty since some samples considered culture-positive showed only one possible pathogen. Culture also reveals many contaminants, which reduces its specificity (35.19%) if these samples are considered false positives. In contrast, PCR exhibits excellent specificity (100%).

Among the diverse diagnostic strategies evaluated, MA showed the best performance, with a sensitivity (98.18%) significantly higher (*p* < 0.001) than strategies based on a single method (78.18% for PCR alone and 74.55% for culture alone) and a specificity similar to PCR (100%). This sensitivity of 98.18% is close to the gold standard defined arbitrarily in this study, although it only requires inoculation of 13% of samples (*K* = 0.98). The MA strategy thus leverages the benefits of DG PCR (enhanced dermatophyte detection, rapid response time, and excellent specificity) while partially mitigating the drawbacks of culture (prolonged and labor-intensive procedures and frequent contamination) by drastically reducing the number of samples necessitating inoculation. This mixed approach, however, would warrant an evaluation of its cost effectiveness.

The study’s limitations primarily stem from methodological considerations in determining fungal pathogenicity. The traditionally accepted rule considers any dermatophyte, regardless of microscopy, as a causative pathogen for onychomycosis, whereas NDM nail infection has to be proven by repeated detection on different samples without concomitant growth of dermatophytes. Nonetheless, this dogmatic rule has recently been questioned by Gupta et al. [34], as repeat sampling is rarely achieved, and their study demonstrates that a combination of NDM detection by PCR and positive histopathology of hyphae may be a good proxy for NDM infection. However, a consensus is still lacking on which cultivated NDM species should be considered contaminants or infectious agents, and most previous studies are not fully transparent about the methodology used to differentiate them. In our study, fungal pathogenicity was determined by arbitrary criteria such as mycelial element visualization on DEC, potential co-detection of clinically significant fungi, and reliance on literature-derived data. For certain fungal species, the availability of such data is currently limited and subject to revision. Although this way of classification remains partly arbitrary and should be considered a limitation, it does, however, allow the reader to assess the relevance of our evaluation, as the results of this classification are presented in Appendix A.

Additionally, it would have been beneficial to gather clinical information including the degree of suspicion for OM, prior treatment history, recurrence rates, or lesion descriptions, given that certain clinical presentations are associated with specific species, particularly melanonychia [35]. Furthermore, the positive PCR results of our study were not confirmed by sequencing technique and could represent false-positive results, but the most likely explanation is the lack of sensitivity of culture, as already mentioned by other authors [30,32]. We can also underline that the MA we propose requires obtaining the best possible results from direct examination to use it effectively as a screening method in combination with PCR, which implies having a well-trained technical team, as the sensitivity of direct examination has been shown to be highly dependent on the operator’s experience [28,32,33,36]. Nevertheless, the use of a fluorochrome such as calcofluor makes the reading easier and more sensitive [32,37,38]. Finally, the sample size precludes an analysis of different strategies in detecting coinfections, although the clinical significance of such occurrences is still poorly elucidated.

## 5. Conclusions

The use of an algorithm that amalgamates the diverse techniques available for detecting onychomycosis pathogens enables the exploitation of their respective advantages while circumventing certain drawbacks. The algorithm assessed in this research exhibits performance akin to the systematic implementation of both techniques on each sample (DG PCR and culture), significantly surpassing the outcomes obtained from a singular technique, although culture is still the only technique offered in most laboratories today. This mixed algorithm obviates the necessity for culture in 87% of instances, leading to substantial time savings in result delivery, combined with the fact that culture is tedious and time-consuming and requires skilled technicians. New generations of multiplex PCR incorporating NDM in their panel could further significantly reduce the number of samples necessitating inoculation.

## Figures and Tables

**Table 1:** Definition of clinical categories assigned to each situation.

Definition of Clinical Categories	Literature: Involvement in OM (Balanced against Commensal Nature and Contaminant Potential)	DEC Positivity (Compatible Image)	Codetection with a Fungus of Greater Clinical Significance
Proven pathogen	Undeniable (dermatophytes)	+/-	yes/no
	Strong	+	no
Probable pathogen	Strong	+	yes
	Moderate	+	no
Possible pathogen	Strong	-	yes/no
	Moderate	+	yes
	Weak	+	no
Probable contaminant	Moderate	-	yes/no
	Weak	+	yes
	None	+	no
Contaminant	Weak	-	yes/no
	None	+	yes
	None	-	yes/no

**Table 2:** Summary of performances of the different diagnostic strategies.

Sample Categorization (n = 106)	Strategies Assessed			Gold Standard
	PCR	Culture	MA	Combinaison
At least one definite pathogen *	43	31	50	50
At least one probable pathogen *	0	4	2	2
At least one possible pathogen *	0	6	2	3
At least one definite, probable or possible pathogen *	43	41	54	55
At least one probable contaminant *	0	10	0	6
At least one definite contaminant	0	19	0	14
Contaminants (probables ou certains)	0	29	0	20
Sterile/not detected	63	36	52	31
Total	106	106	106	106
Sensitivity (definite, probable or possible pathogens)	78.18%	74.55%	98.18%	
Specificity (definite, probable or possible pathogens)	100%	38.60%	100%	
Cohen’s Kappa	0.78	0.74	0.98	
Sensitivity (exclusion of possible pathogens)	82.69%	67.31%	100%	
Specificity (exclusion of possible pathogens)	100%	35.19% *^2^	96.3% *^2^	
Detection of species included in multiplex PCR DG	43	27	46	46
Sensitivity on species included in multiplex PCR DG	93.48% *^3^	58.70%	100%	
Detection of coinfected samples	0	4	3	8

* without codetection of fungus of greater clinical significance. with or without codetection of fungus of lower clinical significance. *^2^ possible pathogens recategorized in false positive samples. *^3^ For 3 samples, a repetition of PCR analysis was needed to highlight the presence of a dermatophyte. With repeated results taken into account, sensitivity of 100%.

## Data Availability

The raw data supporting the conclusions of this article will be made available by the authors on request.

## Appendix A

**Table A1:** List of different fungal species detected and their clinical categorization according to direct examination and possible co-detection of a fungus of greater clinical significance. Occurrences of each case is shown in brackets.

Species Detected (Occurrence in Brackets)	Literature: Clinical Significance in OM	Identification Method		Direct Examination + (Compatible Image)		Direct Examination (or Uncompatible Image)
		Detection by Culture	Detection by DG	Without Coinfection	With Co-Detection of a Fungus of Greater Clinical Significance	
Dermatophytes						
*Trichophyton rubrum* (38)	undeniable	Macro/microscopy (22)	DermaGenius (36)	Proven pathogen (35)		Proven pathogen (3)
*Trichophyton interdigitale* (6)	undeniable	Macro/microscopy (4)	DermaGenius (5)	Proven pathogen (4)		Proven pathogen (2)
Molds						
*Acremonium* (3)	moderate	Macro/microscopy (2) MALDI (1): *Sarocladium kiliense*		-	Possible pathogen (3)	-
*Alternaria* (1)	weak	Reference center (1)		-	Probable contaminant (1)	-
*Arthrinium* sp. (1)	none	Reference center (1)		-	-	Contaminant (1)
*Arthrographis kalrae* (1)	weak	Reference center (1)		-	-	Contaminant (1)
*Aspergillus flavus* (1)	weak (considering contaminant potential)	Macro/microscopy (1)		Possible pathogen (1)	-	-
*Aspergillus fumigatus* (3)	faible (considering contaminant potential)	Macro/microscopy (3)		Possible pathogen (1)	Probable contaminant (1)	Contaminant (1)
*Aspergillus versicolor* (1)	moderate	Macro/microscopy (1)		Probable pathogen (1)	-	-
*Bipolaris* sp. (1)	none	Macro/microscopy (1)		-	-	Contaminant (1)
*Chaetonium* sp. (2)	weak	Macro/microscopy (1) Reference center: sequencing (1)		-	Probable contaminant (1)	Probable contaminant (1)
*Fusarium* sp. (2)	strong	Macro/microscopy (2)		Proven pathogen (1)	-	Possible pathogen (1)
*Neopestalotiopsis* (1)	none	Reference center: sequencing (1)		-	-	Contaminant (1)
*Pénicillium* sp. (12)	none	Macro/microscopy (12)		-	Contaminant (7)	Contaminant (5)
*Phialophora* sp. (2)	none	Macro/microscopy (2) (+1 RNC confirmation)		-	Contaminant (1)	Contaminant (1)
*Scedosporium* sp. (2)	none	Macro/microscopy (2)		-	Contaminant (1)	Contaminant (1)
*Scytalidium dimidiatum* (1)	Strong	Macro/microscopy (1)		Proven pathogen (1)	-	-
*Scopulariopsis* (5)	Strong	Macro/microscopy (5)		Proven pathogen (2)	Probable pathogen (3)	-
*Trichoderma* sp. (1)	none	RNC (1)		-	Contaminant (1)	-
*Ulocladium* (1)	weak	Macro/microscopy (1)		-	Probable contaminant (1)	-
Yeasts						
*Candida albicans* (2)	Strong	MALDI-TOF (1)	DermaGenius (2)	Proven pathogen (2)	-	-
*Candida parapsilosis cplx* (9): *C. parapsilosis* (6) *C. metapsilosis* (2) *C. orthopsilosis* (1)	moderate	MALDI-TOF: *C. parapsilosis* (6), *C. metapsilosis* (2), *C. orthopsilosis* (1)		Probable pathogen (3: *C. parapsilosis*)	-	Probable contaminant (6: 3 *C. parapsilosis* + 2 *C. metapsilosis* + 1 *C. orthopsilosis*)
*Candida pararugosa* (1)	moderate (by extrapolation to other Candida)	MALDI-TOF (1)		-	-	Probable contaminant (1)
*Debaryomyces kansenii* (1)	none	MALDI-TOF (1)		-	-	Contaminant (1)
Yeast not identifiable by MALDI-TOF (2)	moderate (by default)	Macro/microscopy (2)		-	-	Probable contaminant (2)
*Rhodotorula* (4)	none	MALDI-Tof (4)		-	-	Contaminant (4)
*Trichosporon* sp. (7)	weak	MALDI-Tof (7)		-	Probable contaminant (2)	Contaminant (5)
